# The Dual Influence of Video Games on Adolescents' Executive Functions

**DOI:** 10.7759/cureus.76830

**Published:** 2025-01-02

**Authors:** José C Medina-Rodríguez

**Affiliations:** 1 Unidad de Fomento a la Investigación, Instituto Nacional de Psiquiatría Ramón de la Fuente Muñíz, Mexico City, MEX

**Keywords:** child and adolescent psychiatry, executive function, psychiatry and psychology, video game, youth mental health

## Abstract

Video games, defined as interactive digital environments created for entertainment, are becoming increasingly popular among adolescents. These games encompass a wide range, from simple puzzles to intricate multiplayer simulations, each demanding varying degrees of cognitive and motor involvement. Adolescence represents a crucial stage for developing executive functions (EFs), encompassing working memory, inhibitory control, and cognitive flexibility. These advanced cognitive processes are vital for self-regulation, problem-solving, and decision-making. Therefore, the extensive use of video games prompts significant inquiries regarding their developmental effects on youth. Are they beneficial tools for cognitive enhancement, or do they present risks to neurodevelopment? Understanding this complex relationship necessitates an exploration of both the cognitive advantages and potential drawbacks of video gaming. This editorial investigates the impact of video games on EFs in young individuals, emphasizing the necessity for further research to contextualize these effects and formulate evidence-based research methodologies.

## Editorial

Video games are interactive digital environments created for entertainment and have historically been popular among children and adolescents. These games, which range from casual puzzles to complex multiplayer simulations, require developing the nervous system and varying degrees of cognitive and motor dexterity. Adolescence is a critical period for developing executive functions (EFs), including abilities such as working memory, inhibitory control, and cognitive flexibility. These functions are fundamental to self-regulation, problem-solving, and decision-making. This prompts an exploration of whether video games might contribute to universal or clinically relevant outcomes, particularly in this population [1]. Ongoing debates focus on whether current scientific evidence supports the idea that these forms of entertainment can aid as cognitive enrichment tools, or if they may pose risks to developmental processes during youth. Consequently, identifying this reasonable duality necessitates critically evaluating existing medical knowledge and advancing rigorous, high-quality studies addressing these inquiries. Therefore, this editorial aims to provide a straightforward yet objective breakdown of both the positive and negative outcomes associated with gameplay during adolescence. In addition, it situates the found evidence within a broader context to foster innovative research strategies in this area and among this demographic.

First, a meta-analysis supports that specific genres, particularly action and strategy games, have positively affected EFs. Notably, action games, distinguished by their fast pace and, in some conditions, high cognitive demand, may stimulate visuospatial working memory and cognitive flexibility in adolescents. As a consequence, this type of gameplay may moderately contribute to the exercising of multitasking capacities and fit attentional control (Hedges' *g* = 0.49, 95% confidence interval [CI] = 0.35-0.63, *P* < 0.001) [2].

Second, these data coincide with neuroimaging findings, which, although scarce, reveal intensified activation in the prefrontal and parietal cortex during gameplay, regions that are crucial for executive processing and visuospatial functions, respectively. Such stimulation could translate to real-world scenarios requiring rapid problem-solving and adaptability [2]. Therefore, action games may create environments that mandate ongoing aptitude to adapt, reproducing situations where prompt decision-making and flexible thinking are essential, potentially contributing to adequate cognitive competence.

Third, according to a study, courses that employ strategy-based games in educational systems have promoted students' problem-solving abilities. These results may stem from the nature of these genres, which revolve around planning, resource management, and creative decision-making. As such, adolescents who engage in strategy games display moderate but theoretically beneficial changes in these EFs (Hedges' *g* = 0.35, 95% CI = 0.21-0.49, *P* = 0.01) [3].

A study found that adolescents engaging in excessive gaming may experience reduced inhibitory control. Specifically, the findings indicate a moderate effect size (Hedges' *g* = -0.22, 95% CI = -0.30 to -0.14, *P* = 0.01). The paper defines excessive gaming as playing more than 10 hours per week; however, it is not universally agreed upon whether this amount of gaming is beneficial or harmful to adolescents' EFs [4]. One reason for this knowledge gap is that much of the data in this field of research relies on self-reported information, which can introduce biases that, if not carefully addressed, may affect a study's conclusions. Additionally, circadian disruption caused by gaming is another reasonable concern. One study indicates that adolescents who engage in late-night gaming lose 1.5 to 2 hours of sleep. This sleep loss can temporarily alter memory consolidation and attentional control [5]. As such, the study, as mentioned earlier, suggests that youth who prioritize late-night gaming may experience worsened performance in EFs. Nonetheless, while some evidence links gaming to adverse outcomes, further research is still needed.

The data raise an important question: Is the influence of video games on EFs universal or dependent on context? This inquiry is relevant when considering the effects of this activity across different sociocultural and demographic backgrounds. For example, one study focused on adolescents participating in Eastern educational systems found healthy cognitive flexibility and joint problem-solving abilities [3], aligning with the characteristics of collectivist societies. Gaming is occasionally incorporated into the daily curriculum, not only scholarly. Furthermore, it is made clear that gaming is rarely stigmatized in Eastern cultures and is a form of leisure and social activity [3]. Conversely, the study could also propose that such outcomes will hardly be replicated in individualistic Western contexts, especially where gaming sometimes carries a negative social connotation [3]. Moreover, other studies have demonstrated that youth residing in rural areas may opt for simpler gaming genres that can be played offline due to the absence of internet connectivity.

On the other hand, another study suggests that urban adolescents may engage more with online multiplayer games. More importantly, gender disparities in gaming preferences can influence this population's EF outcomes. For instance, the same investigations indicate that males engage more in action and strategy games. In contrast, it also shows that females may prefer puzzle and simulation games [2]. Hence, studying how adolescents play video games can provide valuable information about how it affects their EFs and neurodevelopment, accentuating the need to study adolescent gaming within diverse frameworks to understand their moderating or predicting effects on EFs.

In short, the dual nature of video games' influence on adolescents' EFs requires careful consideration. This area of research is rapidly expanding. Previous yet recent studies often document varied results, implying that accounting for confounding variables can be challenging for researchers. Additionally, the positive and negative effects of gaming on adolescent EFs fall within statistically significant but modest ranges, which calls for cautious deductions about the potential benefits and risks of gaming in this demographic. Examining how sociocultural and demographic differences relate to healthy cognitive growth or potential hazards is essential. Future research should prioritize longitudinal and experimental designs to gain a more profound understanding of these dynamics. Researchers may explore how adolescents engage with video games over time, how distinct genres influence outcomes, or how gaming stigma affects neurodevelopment. Moreover, it is necessary to acknowledge that interpreting the preceding research results is tied to the specific contexts in which the studies were conducted. Finally, it is essential to recall that video games are not recent; they are part of a longstanding tradition enjoyed by individuals of all ages and merit thoughtful discussion.

## References

[1] Shao R, Read J, Behrens TE, Rogers RD (2013). Shifts in reinforcement signalling while playing slot-machines as a function of prior experience and impulsivity. Transl Psychiatry.

[2] Green CS, Bavelier D (2019). The Cognitive Neuroscience of Video Games. The Oxford Handbook of Media Psychology.

[3] Bai Y, Pan W, Hirumi A (2020). A meta-analysis of video games and their impact on cognitive skills in educational settings. J Educ Psychol.

[4] Gentile DA, Choo H, Liau A, Sim T, Li D, Fung D, Khoo A (2011). Pathological video game use among youths: a two-year longitudinal study. Pediatrics.

[5] Cain N, Gradisar M (2010). Electronic media use and sleep in school-aged children and adolescents: a review. Sleep Med.

